# Stigma doesn’t discriminate: physical and mental health and stigma in Canadian military personnel and Canadian civilians

**DOI:** 10.1186/s40359-018-0273-9

**Published:** 2018-12-19

**Authors:** Christine Frank, Mark A. Zamorski, Ian Colman

**Affiliations:** 1 School of Epidemiology, Public Health and Preventive Medicine, University of Ottawa, Ottawa, ON Canada; 2 Department of National Defence, Ottawa, ON Canada; 3 Canadian Forces Health Services Group, Ottawa, ON Canada; 4 Department of Family Medicine, University of Ottawa, Ottawa, ON Canada

**Keywords:** Mental health, Physical health, Enacted stigma, Felt stigma, Stigma, Military, Canadian armed forces

## Abstract

**Background:**

Illness-related stigma has been identified as an important public health concern. Past research suggests there is a disproportionate risk of mental-health stigma in the military, but this same finding has not yet been established for physical-health stigma. The current study aimed to assess the independent contribution of mental and physical health on both enacted stigma (discriminatory behaviour) and felt stigma (feelings of embarrassment) and to determine whether these associations were stronger for military personnel than civilians.

**Methods:**

Data were obtained from the 2002 Canadian Community Health Survey - Mental Health and Well-being and its corresponding Canadian Forces Supplement. Logistic regressions were used to examine a potential interaction between population (military [*N* = 1900] versus civilian [*N* = 2960]), mental health, and physical health in predicting both enacted and felt stigma, with adjustments made for socio-demographic information, mental health characteristics, and disability.

**Results:**

Mental health did not predict enacted or felt stigma as a main effect nor in an interaction. There was a strong link between physical health and enacted and felt stigma, where worse physical health was associated with an increased likelihood of experiencing both facets of stigma. The link between physical health and enacted stigma was significantly stronger for military personnel than for civilians.

**Conclusions:**

Physical health stigma appears to be present for both civilians and military personnel, but more so for military personnel. Elements of military culture (e.g., the way care is sought, culture of toughness, strict fitness requirements) as well as the physical demands of the job could be potential predictors of group differences.

## Background

Illness-related stigma has been identified as an important public health concern [1, 2], with many documented negative effects including anxiety [3], stress [4], depression [5], reduced self-esteem/self-efficacy [6], reduced or delayed care-seeking [7, 8], and lowered adherence to treatment [9].

There are multiple ways to conceptualize stigma. Link and Phelan [10] argue that, due to the complexities of stigma as a construct, it is important to allow variation as long as a clear definition of stigma is provided by the researchers. In this research, we are drawing on the multi-layered definition of stigma outlined by Scambler and Hopkins [11] who suggest there are two facets of stigma: enacted stigma and felt stigma. Whereas enacted stigma refers to the perceived act of discrimination against individuals with a stigmatizing condition, felt stigma refers to the individual’s embarrassment and shame associated with the condition. This conceptualization allows for a multifaceted assessment of stigma by including both behaviours towards the individual, and feelings of the individual. Both mental and physical health problems can lead to enacted or felt stigma, though mental disorder-related stigma has been a particular focus recently, with major organizations such as the World Health Organization identifying stigma reduction as a key target for population mental health strategies [12].

The associations between felt and enacted stigma and mental health issues have been documented in many domains, including access to health care, housing, intimate relationships, and employment [13–15]. Findings have consistently shown that those who have mental health conditions are at increased risk of discrimination and negative feelings [16–18]. Findings have also similarly linked physical health problems (e.g., epilepsy, HIV, obesity) with stigma, where those with physical health conditions have a higher likelihood of experiencing discrimination and embarrassment [19–22]. Health-related stigma is strongly related to one’s social environment and, for employed individuals, the workplace is a crucial part of one’s social environment. In the workplace health-related stigma is associated with a lack of career advancement, poor quality of work, as well as diminished employability, and increased likelihood of being unemployed or under-employed [23, 24].

Military organizations are large employers, and their personnel fulfill crucial functions in the protection of national interests and promotion of international peace and security. The mental health of military personnel has attracted attention over the past 15 years, as a result of the deployment of millions of Western military personnel in support of the conflicts in Southwest Asia [25] as well as peacekeeping missions in Africa, Asia, and South America [26]. The impact of such deployments on mental health have been substantial. Not only are mental health issues more prevalent in the military than in the general public [27–31], but a recent study found the prevalence rates of mental health issues in the Canadian Armed Forces (CAF) have increased over the past 10 years, with significant increases in post-traumatic stress disorder (PTSD), general anxiety disorder, and panic disorder [32]. Being in the CAF also has a significant impact on a member’s physical health. Indeed, those in the military have a higher risk of experiencing training- or deployment-related injuries or illnesses, such as musculoskeletal injuries [33], traumatic brain injury [34, 35], or tinnitus/hearing loss [36].

A disproportionate burden of mental disorder-related stigma has been posited in military personnel [37]. The same factors that explain why a disproportionate risk of mental-health stigma may exist also relate to why there may be a disproportionate risk of physical-health stigma. First, the armed forces have strict fitness and health standards. Those with restrictions related to physical or mental health may be deemed unfit for promotion or continued service, or unable to go on course or deploy [38–41]. This impact to their professional development may be perceived as discriminatory by the person in question. Additionally, in the CAF, both physical and mental health care is provided by the employer, which means there is an increased risk of having one’s superiors find out about one’s health status (e.g., if an individual needs to be sent home for a medical reason while on training or deployed). Confidentiality issues appear to be a top concern for members as a recent qualitative study examining barriers to care among military health care providers found concerns about confidentiality was one of the top system-level barriers [42]. Also, there is a general focus on being strong and tough within the military [43], which may enhance negative opinions of those who have a physical or mental health issue and are no longer able to do the same tasks they were once able to do. Last, due to the high physical and mental demands of the job and the strict fitness and health standards, physical and mental health issues have a substantial impact on job performance [44]. This may be problematic, as a study by McLaughlin, Bell, and Stringer [45] found work impact was the only significant predictor among a set of variables (e.g., onset controllability, social impact of disability) to consistently predict stigma and acceptance. That is, the more one’s health issue impacted one’s work, the more stigma and less acceptance were reported by colleagues.

Empirical evidence of the excess burden of stigma in military personnel relative to civilians had been limited until the recent publication of findings showing CAF military personnel were 1.7 times more likely to have mental health-related stigma relative to a comparable civilian sample, even after careful adjustment for the important differences in sociodemographic and need-related factors between the populations [37]. Additionally, CAF personnel also reported perceived stigma had more negatively affected their workplace experience compared to civilians. However, the analyses by Weeks and colleagues [37] did not distinguish between felt and enacted stigma and only looked at stigma related to mental health problems (and not physical health problems).

Research has yet to examine whether these same group differences emerge when assessing physical health-related stigma. In fact, very little research has been conducted to examine the impact of physical health issues on stigma in military populations. One study of United States (U.S.) soldiers returning from Bosnia suggested that many soldiers believed admitting a physical health issue would result in stigma, with 43% of the soldiers agreeing that admitting a physical issue would harm their career and 22% believing that admitting a physical health issue would cause their friends to distance themselves [46]. To our knowledge, no studies have explicitly explored differences between military personnel and civilians on physical health-related stigma. This is important as destigmatization messages in military populations have sought to reframe mental health problems as analogous to physical injuries, for example using the term operational stress injury [47]. Given this, it is important to understand whether a relationship between physical health and stigma exists in the military and whether the association is stronger for those in the military compared to civilians.

Given that both mental and physical health issues are related to the experience of enacted and felt stigma, there may also be an additive effects, should an individual experience both poor mental health and poor physical health. However, very little research to date has looked at the potential interaction between physical and mental health in relation to the experience of stigma and no research has looked at this potential interaction by population. One study in the general population found that perceived stigma was higher for those who had both a physical illness and a psychiatric illness compared to those who only had a psychiatric illness, offering some support for the supposition that physical health may contribute incrementally (in additive or interactive ways) to the prediction of stigma [48].

### Current study

The goal of this study was to replicate and expand on past research examining stigma and health. To do this, we used a comparable sample of civilians and military personnel to:

1. Determine whether there is a risk of stigma related to physical health;
2. Determine whether there is a disproportionate risk of physical health stigma in the military compared to civilians;
3. Assess the relative contribution of both physical and mental health on the likelihood of experiencing enacted and felt stigma; and
4. Determine whether there is an interaction between physical health, mental health and population (military versus civilians). More specifically, whether the two-way interaction between mental and physical health is stronger among military personnel compared to civilians.

## Method

### Data source

Data came from the 2002 Canadian Community Health Survey Cycle 1.2 – Mental Health and Well-being (CCHS-MH Civilian) and its corresponding Canadian Forces Supplement (CCHS-MH Military) [49]. Both surveys employed a sampling framework, resulting in representative samples of CAF personnel and the Canadian general population.

Statistics Canada interviewers collected the data using a computer-assisted, face-to-face interview, and the wording of all overlapping content across surveys was identical [49, 50]. In terms of survey coverage, the CCHS-MH Military included a total of 5155 CAF Regular Force personnel (response rate = 79%) [50].

The CCHS-MH Civilian included individuals aged 15 and older living in private dwellings in the 10 provinces, excluding individuals living in the three territories, reserves, or on Crown Lands, full-time members of the CAF, and the institutionalized population (exclusions represent about 2% of the target population) [49]. A total of 36,984 individuals (for an individual response rate of 89.0%) provided responses for the survey. We followed procedures from two recent papers to restrict the civilian sample in order to more closely match the socio-demographic and health characteristics of the military population [30, 37]. Our matched civilian sample included only those who: 1) were full-time employed; 2) were aged 17 to 60 (the age range of the military sample); 3) had not immigrated in the past 5 years (who were therefore not eligible for citizenship and hence, military service); and 4) had not reported any chronic conditions that would typically preclude military service (e.g., heart disease, severe obesity) [30].

The survey assessed both enacted and felt stigma using items that were part of the Restriction of Activity module (see below). Specifically, respondents who either indicated having had any difficulty “hearing, seeing, communicating, walking, climbing stairs, bending, learning or doing any similar activities”, or indicated a “long-term physical condition or mental condition or health problem” that reduced the amount or the kind of activity they can do in four domains (i.e., home, work, school, other) completed the Restriction of Activity module. Only those who completed the Restriction of Activity module were included in this study. Our final sample included 1900 members from the CAF and 2960 civilians.

### Measures

#### Enacted stigma

Enacted stigma was assessed by asking respondents to indicate how much discrimination or unfair treatment they experienced due to a physical or mental condition or health problem over the past 12 months (1 = “none at all”, 2 = “a little”, 3 = “some”, or 4 = “a lot”). Due to extreme skew identified during data cleaning (93.51% of the civilian sub-sample and 83.78% of the military sub-sample reported experiencing no stigma related to their condition in the past 12 months), the item was dichotomized (experienced enacted stigma: yes/no) as suggested by MacCallum, Zhang, Preacher, and Rucker [51] as an appropriate solution. This solution also addressed the issue of having a limited number of responses in the “a lot” category.

#### Felt stigma

Felt stigma was assessed by asking respondents to indicate how much embarrassment they experienced due to a physical or mental condition or health problem over the past 12 months (1 = “none at all”, 2 = “a little”, 3 = “some”, or 4 = “a lot”). Similar to enacted stigma, felt stigma was also extremely skewed (80.42% of the civilian sub-sample and 77.75% of the military sub-sample reported experiencing no embarrassment due to their condition in the past 12 months) and had limited responses in the “a lot” category. Thus the responses were also dichotomized (experienced felt stigma: yes/no).

#### Physical health

Physical health was assessed using a single self-report item that asked respondents “In general, would you say your physical health is: poor, fair, good, very good, or excellent” [52]. Higher scores indicate better perceived physical health. Research has shown this item to have a robust association with more objective health outcomes, including obesity [53], cardiovascular disease [54], diabetes [55], mortality [56], and use of health services [57]. The single-item physical health question has been identified as being appropriate for use in population surveys [58].

#### Mental health

Mental health was assessed using a single self-report item that asked respondents “In general, would you say your mental health is: poor, fair, good, very good, or excellent” [52]. Higher scores indicate better perceived mental health. A meta-analytic review of the usage of the single item indicated the item correlated moderately with the Kesseler Psychological Distress Scale (K10), the Patient Health Questionnaire, the mental health subscales of the Short-Form Health Status Survey, and increased health service utilization [59].

#### Socio-demographic characteristics

Socio-demographic variables included sex, age, ethnicity (white or non-white), marital status (single, separated/divorced/widowed, or married/common-law), income adequacy (low income [< $15,000 if 1 or two people; < $20,000 if 3 or 4 people; < $30,000 if 5+ people] or middle-high income [≥ $15,000 if 1 or 2 people; ≥ $20,000 if 3 or 4 people; ≥ $30,000 if 5+ people]), and highest educational attainment (less than secondary [high] school graduate, secondary school graduate, some post-secondary education, and post-secondary diploma or degree).

#### Mental health characteristics

We used several measures common to both surveys to control for differences in mental health in the two populations.

#### Mental disorders

The World Health Organization Composite International Diagnostic Interview (WHO-CIDI 2.1) [60] was used to assess the presence of past-year mental disorders. The following disorders were measured against Diagnostic and Statistical Manual of Mental Disorders-IV (DSM-IV) criteria in both surveys: major depressive episode, panic disorder, and social phobia.

#### Alcohol dependence

Alcohol dependence was measured using a subset of items from the Composite International Diagnostic Interview (CIDI) developed by Kessler and Mroczek [61]. Respondents were asked to respond either yes (scored as 1) or no (scored as 0) to nine alcohol-related questions (e.g., during the past 12 months, have you ever been drunk or hung-over while at work, school or while taking care of children). Respondents were either classified as low risk (scores of 0–2) or high risk (scores of 3–7) for alcohol dependence.

#### Suicidal ideation

Suicidal ideation was assessed by asking respondents whether they had “seriously thought about committing suicide or taking [their] own life” in the past 12 months.

#### Psychological distress

The K-10 [62] was used to assess overall levels of psychological distress experienced during the past 30 days. The 10 items were rated on a 5-point scale and summed to create a total distress score from 0 to 40, with higher scores indicating higher levels of mental illness symptoms. For the current study, we trichotomized distress scores based on cut-offs reported in Australian population research [63]: “low” (0–5), “moderate” (6–19), and “high” (20–40).

#### Disability

Severity of disability was measured using two items. The first item asked respondents to report how many days over the past 2 weeks they had to stay in bed at all because of illness or injury. The second question asked respondents how many days over the past 2 weeks they had to reduce the number of things they normally did because of illness or injury. Responses on both items ranged from 0 to 14 days. Both items were included as independent predictors of stigma.

### Analysis

To assess our objectives, two sets of hierarchical logistic regressions were conducted using Stata version 13.1, with enacted stigma and felt stigma as the outcomes (presence of stigma = 1, absence of stigma = 0). All analyses were conducted using survey and bootstrap weights generated by Statistics Canada, making the samples representative of the source populations. Weights provided by Statistics Canada capture the complex sampling scheme and non-response adjustments. Variance was estimated using bootstrap methods using replicate weights also provided by Statistics Canada.

For both sets of analyses, the first model included population (civilian or military), physical health, mental health, and all 2-way and 3-way interaction terms (i.e., a physical health by population interaction term, a mental health by population interaction term, physical health by mental health interaction term, and the population by physical health by mental health interaction term). In the second model, all socio-demographic variables were added (sex, age, marital status, income adequacy, education, ethnicity). In the third and final step, mental health variables and disability were added to the model (depression, panic disorder, social phobia, distress alcohol dependence, suicidal ideation). The *margins* command in Stata [64] was used to assess whether there were statistically significant differences between the groups of interest and to compare the predicted probabilities across groups.

Due to unexpected results relating to the lack of association between mental health and both enacted and felt stigma, a post-hoc analysis was also conducted to examine how respondents responded to an item asking them to indicate the main cause of their health condition (i.e., which one of the following is the best description of the cause of this condition).

## Results

Socio-demographic and health information for the two populations is outlined in Table 1. Of note, the military sub-sample had a higher prevalence of males than the civilian sub-sample, as well as a higher prevelance of middle aged, white, and married individuals. Military personnel were more likely to report experiencing enacted stigma with 16.34% (95% C.I. [14.44; 18.24]) indicating they had experienced discimination over the past 12 months compared to 6.50% of civilians (95% C.I. [5.39; 7.61]). Military personnel were equally likely to report experiencing felt stigma, with 22.23% reporting having experienced feelings of embarrassment over the past 12 months (95% C.I. [20.15; 24.31]) compared to 19.58% of civilians (95% C.I. [17.63; 21.52]).

**Table 1:** Prevalence of socio-demographic characteristics among military personnel and civilians

Characteristics	Civilian Sub-Sample (*N* = 2960)	Military Sub-Sample (*N* = 1900)
	% [95% C.I.]	% [95% C.I.]
Sex		
Male	59.85 [57.44; 62.26]	89.23 [88.48; 89.98]
Female	40.15 [37.74; 42.56]	10.77 [10.02; 11.52]
Age group, years		
< 25	11.67 [10.13; 13.21]	6.06 [4.76; 7.36]
25–34	17.77 [15.83; 19.72]	25.41 [23.31; 27.51]
35–44	32.18 [29.77; 34.59]	52.36 [49.89; 54.83]
> 44	38.37 [35.92; 40.82]	16.17 [14.55; 17.79]
Ethnicity		
White	86.23 [84.11; 88.35]	95.17 [94.11; 96.24]
Non-white	13.77 [11.65; 15.89]	4.83 [3.76; 5.89]
Marital status		
Single	22.00 [20.11; 23.90]	14.93 [13.04; 16.82]
Married/Common-law	69.26 [67.02; 71.49]	75.14 [72.97; 77.32]
Widowed/Separated/Divorced	8.74 [7.31; 10.17]	9.92 [8.39; 11.45]
Income Adequacy		
Low Income	4.92 [3.94; 5.91]	0.19 [0.05; 0.34]
Middle or High Income	95.08 [93.82; 96.33]	99.81 [99.29; 100.32]
Highest education attained		
Less than secondary	14.72 [12.89; 16.55]	8.48 [7.10; 9.85]
Secondary	20.72 [18.59; 22.86]	33.91 [31.52; 36.30]
Some post-secondary	9.03 [7.55; 10.50]	13.29 [11.62; 14.97]
Diploma or degree	55.53 [52.92; 58.14]	44.32 [41.98; 46.64]
Physical Health		
Poor	2.99 [2.00; 3.98]	5.19 [4.06; 6.33]
Fair	13.34 [11.55; 15.13]	15.67 [13.81; 17.54]
Good	40.40 [37.48; 42.99]	38.08 [35.63; 40.53]
Very Good	32.91 [30.48; 35.34]	32.50 [30.17; 34.83]
Excellent	10.36 [8.67; 12.06]	8.56 [7.27; 9.85]
Mental Health		
Poor	1.34 [0.91; 1.77]	3.85 [2.89; 4.81]
Fair	7.26 [6.01; 8.50]	10.40 [8.88; 11.93]
Good	32.16 [29.77; 34.55]	31.70 [29.44; 33.95]
Very Good	37.14 [34.51; 39.76]	39.59 [37.11; 42.08]
Excellent	22.11 [19.58; 24.64]	14.45 [12.70; 16.20]
Major Depressive Episode	6.66 [5.60; 7.71]	12.00 [10.40; 13.61]
Panic Disorder	2.55 [1.82; 3.27]	2.78 [1.95; 3.60]
Social Phobia	5.00 [3.77; 6.22]	6.03 [4.86; 7.19]
Suicidal Ideation	5.32 [4.30; 6.35]	5.79 [4.71; 6.87]
Alcohol Dependence		
Low Risk	4.60 [3.64; 5.56]	4.74 [3.51; 5.97]
High Risk	95.40 [94.44; 96.36]	95.26 [94.03; 96.49]
Psychological Distress		
Low Risk	51.78 [49.20; 54.37]	57.23 [54.80; 59.66]
Moderate Risk	44.65 [42.04; 47.25]	38.82 [36.45; 41.20]
High Risk	3.57 [2.95; 4.95]	3.95 [2.95; 4.95]

First, we tested whether the models predicted enacted stigma. Results indicated a significant main effect of population, where those in the military were more likely to report enacted stigma compared to civilians (OR = 5.95, 95% C.I. [1.67; 21.09]) and a significant interaction between physical health and population (OR = 0.52, 95% C.I. [.27; .99]). The interaction between mental health and population, as well as the interaction between mental health and physical health were not significant. Additionally, the three-way interaction between population, mental health, and physical health was also not significant (see Table 2).

**Table 2:** The effect of military (versus civilian) and perceived physical health on enacted stigma

Predictor	Model 1			Model 2^a^			Model 3^b^		
	O.R.	95% C.I.	*p*	O.R.	95% C.I.	*p*	O.R.	95% C.I.	*p*
Population	6.67	[2.17; 20.51]	.001	5.70	[1.73; 18.88]	.004	5.95	[1.67; 21.09]	.006
Physical Health	0.89	[0.52; 1.51]	.66	0.81	[0.47; 1.40]	.46	0.86	[0.50; 1.49]	.59
Mental Health	0.79	[0.53; 1.19]	.26	0.78	[0.51; 1.19]	.25	0.84	[0.61; 1.45]	.79
Population x Physical Health	0.47	[0.26; 0.87]	.02	0.52	[0.28; 0.98]	.04	0.52	[0.27; 0.99]	.05
Population x Mental Health	0.85	[0.52; 1.39]	.52	0.87	[0.53; 1.45]	.60	0.87	[0.52; 1.46]	.61
Mental Health x Physical Health	0.91	[0.74; 1.11]	.34	0.93	[0.76; 1.14]	.50	0.92	[0.75; 1.13]	.43
Population x Mental Health x Physical Health	1.19	[0.93; 1.51]	.15	1.16	[0.91; 1.48]	.24	1.17	[0.91; 1.50]	.21

^a^Adjusted for socio-demographic characteristics: sex, age, marital status, education, ethnicity, income

^b^Adjusted for socio-demographic characteristics, disability, and mental health: depression, distress, alcohol dependence, panic disorder, social phobia and suicidal ideation

**Table 3:** The effect of military (versus civilian) and perceived mental health on felt stigma

Predictor	Model 1			Model 2^a^			Model 3^b^		
	O.R.	95% C.I.	*p*	O.R.	95% C.I.	*p*	O.R.	95% C.I.	*p*
Population	1.98	[0.62; 6.26]	.25	1.80	[0.56; 5.74]	.32	1.57	[0.52; 4.74]	.43
Physical Health	0.64	[0.43; 0.96]	.03	0.62	[0.41; 0.93]	.02	0.65	[0.42; 0.98]	.04
Mental Health	0.79	[0.51; 1.22]	.28	0.74	[0.49; 1.12]	.15	0.76	[0.51; 1.14]	.18
Population x Physical Health	0.88	[0.52; 1.50]	.65	0.90	[0.53; 1.53]	.70	0.85	[0.50; 1.43]	.53
Population x Mental Health	0.87	[0.49; 1.55]	.64	0.93	[0.91; 1.25]	.40	1.04	[0.64; 1.70]	.87
Mental Health x Physical Health	1.05	[0.80; 1.25]	.97	1.07	[0.91; 1.25]	.84	1.07	[0.91; 1.26]	.39
Population x Mental Health x Physical Health	1.00	[0.37; 2.26]	.85	0.98	[0.79; 1.21]	.84	0.99	[0.80; 1.21]	.89

^a^Adjusted for socio-demographic characteristics: sex, age, marital status, education, ethnicity, income

^b^Adjusted for socio-demographic characteristics, disability, and mental health: depression, distress, alcohol dependence, panic disorder, social phobia and suicidal ideation

**Fig. 1:**
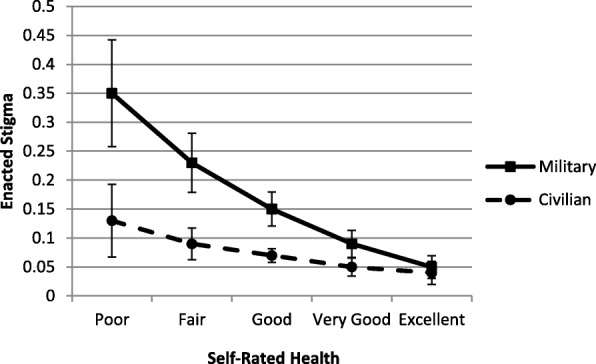
Predicted probability of enacted stigma across health for the two populations

Adjusted predicted probabilities were calculated to explore the interaction between military/civilian status and physical health. Among both groups, as physical health increased, the likelihood of stigma decreased, but the strength of this relationship significantly differed by military/civilian status, *B* = −.05, *SE* = .01, *p* < .001, 95% C.I. [−.07; −.03]. The negative link between physical health and enacted stigma was much stronger for military personnel, *B* = −.07, *SE* = .01, *p* < .001, 95% C.I. [−.09; −.05], than civilians, *B* = −.02, *SE* = .01, *p* = .01, 95% C.I. [−.03; −.004]. Absolute adjusted risk differences were calculated at each level of health, revealing differences between the two populations were largest at poor physical health, decreasing as physical health improved until no significant difference was observed at excellent health (see Fig. 1).

Next, we tested whether the same models predicted felt stigma. Only physical health was a significant predictor of felt stigma, where better physical health was related to a lower likelihood of felt stigma (OR = 0.65, 95% C.I. [.42; .98]). Again, the interaction between mental health and population, as well as the interaction between mental health and physical health,^1^ were not significant. Additionally, the three-way interaction between population, mental health, and physical health was also not significant (see Table 3).

### Post-hoc analysis

Due to the unexpected findings that mental health did not significantly predict enacted or felt stigma in our multivariate models, we conducted a post-hoc examination to examine how respondents responded to a question assessing the cause of their health problem (this would be the same health problem referenced for both stigma items). We noted that only 3.47% of the military sub-sample and 4.05% of the civilian sub-sample identified emotional or mental health as the cause for their illness. In the civilian population, disease or illness (26.39%), birth condition (12.40%), and work condition (12.25%) were the three most common causes of the health condition. In the military population, accident at work (29.60%), work conditions (26.23%), and disease or illness (12.63%) were the most common causes of the health condition.

## Discussion

This study assessed whether there was an association between physical health and the experience of enacted and felt stigma and whether this association was stronger among military personnel than civilians. The study also attempted to examine the relative impact of mental health and physical health on enacted and felt stigma. Last, the study explored the potential interaction between population (military versus civilians), physical health, and mental health in predicting enacted and felt stigma.

First, when assessing the association between physical health and stigma, the results showed a link between physical health and both enacted and felt stigma, where worse health was linked to an increased probability of experiencing felt and enacted stigma. This finding replicates past research that linked stigma and specific physical disabilities [20, 21, 65], and points to the existence of general physical health stigma. Furthermore, the population (military versus civilians) by physical health interaction was a significant predictor of enacted stigma, but not felt stigma. The link between physical health and enacted stigma was stronger for military personnel than for civilians, even after adjusting for differences in socio-demographics, mental health, and severity of disability. Differences in predicted probability of experiencing enacted stigma between military personnel and civilians were most pronounced when physical health was poor, with differences between the two populations decreasing as physical health improved. In other words, as health worsens, both groups have an increased probability of experiencing perceived discrimination, but this increased risk is amplified for military personnel. This pattern of findings expands on past research showing differences in reported stigma among military personnel and civilians [37]. While it is clear there is a difference in risk of health-related stigma between military personnel and civilians, it is currently unclear what drives this difference. For one, there may be factors inherent to the military environment. As mentioned earlier, those with physical or mental health issues in the military commonly (or exclusively for regular force members) seek care through their employer (the CAF). Due to this, their chain of command may become aware of their health issues through either a ‘need to know’ or through a breach in confidentiality. In their qualitative review Born and colleagues [42] found that health care providers reported seeing confidentiality breaches among the health care staff (regarding both their own information as well as other patient’s information). In turn, this may result in not being promoted, sent on course, or deployed, which could be perceived as discriminatory by the individual [38–41]. Additionally, the ‘culture of toughness’ in the military may contribute to this difference, as those who are unable to do the physical tasks they once were able to do prior to an injury or illness may be perceived as weak or less valuable to the organization/team. Second, the physical demand of the employment is potentially a factor. As noted, the military has high physical standards for service. Physical health issues may result in the member no longer being eligible for service and being medically discharged, even if their particular trade does not require a high level of physical activity, which also may be seen as discriminatory by the member. Additionally, for some trades (e.g., infantry) their job is physically demanding. As such, for those individuals injury or a decline in physical health may be much more detrimental to employment than it would be in professions that are much less physically demanding (e.g., office jobs). Previous research has found that job performance is a strong and significant predictor of acceptance in the workplace [45]. In future research, it is important to determine what factors are driving these group differences. Although physical health significantly predicted felt stigma, this association did not appear to be modified by population (civilian versus military). It may be that the association, while present, is simply weaker.

We also aimed to examine the relative influence of physical health issues and mental health issues on enacted and felt stigma. Results of our multivariate model suggest that, in both military and civilian populations, physical health has a strong association with enacted and felt stigma, whereas mental health does not. However, rather than reflecting reality, these findings more likely reveal a limitation of our stigma items. To better understand the results, we reexamined the stigma questions to assess whether the items were equally reflecting discrimination and embarrassment relating to physical conditions *and* mental health conditions (as the item prompt refers to both). First, we examined the questions from the Restriction of Activities section that directly preceded the enacted and felt stigma items and found the phrasing of the questions appeared more applicable to physical health conditions than mental health conditions (e.g., do you have any difficulty hearing, seeing, communicating, walking, climbing stairs, bending, learning or doing any similar activities?; does a long-term physical condition or mental condition or health problem, reduce the amount or the kind of activity you can do?). It is possible, given the phrasing of the questions, respondents were primed to refer to experiences relating to physical and not mental health conditions. As outlined in the results, we also examined responses to a question that assessed the cause of respondents’ health problem and noted very few individuals (< 5%) identified emotional or mental health as the cause for their illness. With these findings, we determined it was reasonable to conclude that most respondents were likely reporting on experienced stigma associated with a physical health condition and not a mental health condition. This would explain why there was such a strong effect of physical health on stigma and no significant effect of mental health, despite a substantial amount of research showing a link between mental health and stigma in both civilian and military populations [16–18, 37, 46, 48]. Moving forward, it will be important to test this hypothesis using a more suitable dataset that captures stigma related to mental and physical health problems either separately, or at least more equally.

We also explored a potential three-way interaction between physical, mental health, and population. This interaction was not significant in either of the analyses. Again, given the substantial amount of research that has shown a link between mental health and stigma [16–18, 37, 46, 48], it is unlikely that our findings represent a true pattern in the data. Rather, findings are more likely due to the failure of our stigma items to tap into mental health related stigma, as discussed previously.

### Implications

One of the key implications of the findings is that increased awareness of physical health-related stigma is important. As mentioned in the introduction, there is currently a shift to ‘re-brand’ psychological issues as medical issues in the military (e.g., “Illness like any other”). As an example, labelling psychological issues stemming from duty as ‘operational stress injuries’ is seen as a way to give psychological issues the same legitimacy as medical issues. It is perceived as a way to de-stigmatize mental health problems in the CAF [47]. However, if there is stigma associated with physical health conditions, as the present results suggest, this strategy may not be particularly effective. It appears it may be more beneficial to focus efforts on reducing the stigma related to all health issues.

These findings may also have implications for how to approach stigma associated with mental health issues. In recent years, there has been a focus on reducing mental health stigma in both the military (e.g., in Canada, “The Road to Mental Readiness” campaign and, in the U.S., the “Real Warriors. Real Battles. Real Strength” campaign) and the general population (e.g., in Canada, the “Bell’s let’s talk campaign” and, in the U.S., the “Bring Change 2 Mind” campaign). Our findings suggest it may be valuable to combine efforts and focus on reducing stigma related to all health issues instead of targeting only psychological health issues.

### Limitations & strengths

First, and perhaps most importantly, as outlined in the main discussion, it appears that the items measuring enacted and felt stigma were not tapping into both mental health-related and physical health-related stigma, but predominantly physical health-related stigma. However, the value of the findings showing that physical health is linked to enacted and felt stigma and that this association differs by population (civilians and military personnel) should not be diminished. It is important, to identify the factors that predict this excess burden of physical health stigma in the military and expand on past physical health and stigma research by identifying potential modifiers of the relationship.

Because the data are cross-sectional, we cannot with certainty infer causality. It may not be that those with worse physical health experience worse discrimination and embarrassment, but that individuals with worse physical health are more likely to self-stigmatize, resulting in increased embarrassment as well as the increased perception of discrimination. A study by Jones and colleagues [41] indicated that stigma is dynamic and varies with the intensity of mental health symptoms. Because the stigma items referred to felt or enacted stigma experienced over the past 12 months and the mental and physical health assessments referred to current health, another possibility is that the experience of felt stigma actually could lead to worse mental health. For example, it may be that one’s feelings of embarrassment about one’s condition leads to a delay in treatment which is related to worse outcomes [66]. If the hypothesized relation actually exists in reverse (or is bidirectional), it may be more beneficial for stigma campaigns to also focus on reducing stigma at both individual and organizational levels.

Another limitation of the study is the age of the data. Both the civilian and the military data were collected in 2002, which was 15 years ago. One might argue that the findings of this data may no longer be applicable, but this is likely not the case. First, as previously mentioned, both public and military policies and interventions have focused specifically on mental health stigma. No work had been done to decrease physical health stigma, suggesting that it is likely still an issue today. Furthermore, even with campaigns working to reduce mental health stigma in the military, more current data (collected in 2012) has still found a higher burden of stigma (mental health stigma) in the military compared to civilians [37]. If mental health stigma is still problematic despite campaigns targeting stigma reduction, it is likely that physical health stigma is still an issue.

Last, we only have a single-item broad measure to assess physical health. A limitation of the single item is that it is possible that different types of physical conditions, or, different aspects of physical conditions (e.g., reduced mobility, shortness of breath, muscle weakness) have different relationships with stigma (discussed in future directions) which we are not able to test. However, this may not be problematic, as a literature review on physical disabilities and stigma conducted by van Brakel [65] concluded that the impact of stigma was similar across disabilities. Additionally, recently, Hatzenbuehler, and colleagues [1] suggested it may be hard to assess the true magnitude of the relationship between physical health and stigma because studies examining the link have been compartmentalized into separate domains (e.g., stigma and obesity, stigma and HIV). and suggest that research broaden its scope to examine a more general conceptualization of physical health and stigma.

This study also had a number of strengths. For instance, we used data from two concurrent, population-based surveys, increasing the reliability of our results. Also, we employed robust methodological procedures, including sample restriction to create a sample of Canadian civilians that was more comparable to the military population as well as adjustments for key socio-demographic characteristics, variables related to the need for mental health care, and disability.

## Conclusion

Stigma, it seems, does not discriminate with respect to the nature of the health problem (mental versus physical). The findings suggest that, as physical health worsens, the risk of experienced discrimination and embarrassment increases. Furthermore, the increase in probability of enacted stigma is particularly problematic in the military, where the association is significantly stronger than in the general population. The findings suggest future stigma reduction campaigns should consider including physical health stigma as well as mental health stigma. Future research should examine what factors contribute to physical health stigma, and identify whether certain aspects of poor physical health modify the link between physical health and stigma.

## Abbreviations

CAF: Canadian Armed Forces
CCHS-MH: Canadian Community Health Survey- Mental Health
CI: Confidence interval
CIDI: Composite international diagnostic interview
PTSD: Post-traumatic stress disorder
U.S.: United States
WHO-CIDI: World Health Organization Composite International Diagnostic Interview

## Footnotes

1 Adjusted predicted probabilities were calculated for the non-significant interaction between military/civilian status and physical health. For both groups as physical health increased the likelihood of felt stigma decreased. The strength of this relation only marginally differed by population where the association between health and felt stigma was marginally stronger for military personnel, *B* = −.07, *SE* = .01, *p* < .001, 95% C.I. [−.09; −.04], than for civilians, *B* = −.04, *SE* = .01, *p* = .004, 95% C.I. [−.06; −.01].
